# Daptomycin‐induced hyperkalemia: A case report and brief description of mechanism

**DOI:** 10.1002/ccr3.8188

**Published:** 2023-11-12

**Authors:** Praveen Errabelli, Maulik Lathiya, Sasmit Roy

**Affiliations:** ^1^ Nephrology Department Mayo Clinic Health Systems Eau Claire Wisconsin USA; ^2^ Emergency Department Mayo Clinic Health Systems Eau Claire Wisconsin USA; ^3^ Department of Nephrology Centra Lynchburg General Hospital Lynchburg Virginia USA

**Keywords:** acute kidney injury, Daptomycin, hyperkalemia, rhabdomyolysis

## Abstract

**Key Clinical Message:**

Daptomycin causes serious side effects like rhabdomyolysis at high doses. At lower doses it can cause isolated hyperkalemia without frank rhabdomyolysis. Checking BMP along with CK helps taking timely measures to prevent adverse consequences.

**Abstract:**

Hyperkalemia is a common yet challenging clinical condition faced daily by physicians worldwide. Accurate etiology and timely management are paramount in correcting this preventable yet life‐threatening electrolyte imbalance. Very seldom has Daptomycin been implicated as a culprit for hyperkalemia. We present one such unique case where a low dose of Daptomycin led to hyperkalemia, and timely identification improved patient outcomes. We present a 69‐year‐old woman with multiple comorbidities admitted to the intensive care unit to manage diabetic ketoacidosis and sepsis. She developed acute kidney injury due to intravenous contrast, volume depletion, and obstructive uropathy. Interestingly although initially normokalemic, as her renal function started improving with sound urine output, she developed recurrent hyperkalemia, which required medical management. The etiology of hyperkalemia was initially unclear, but on closer review, it was discovered that Daptomycin was the potential culprit. Although case studies with high‐dose Daptomycin causing rhabdomyolysis and hyperkalemia have been reported, low‐dose Daptomycin causing hyperkalemia without rhabdomyolysis has never been reported, bringing forth the uniqueness of our article.

## 1 INTRODUCTION

Daptomycin is a cyclic lipopeptide antimicrobial used to treat complicated gram‐positive infections, including those infected with methicillin‐resistant Staphylococcus aureus. It is typically dosed at 4–6 mg/kg body weight/day. Common side effects include anemia, diarrhea, nausea, vomiting, and rarely rhabdomyolysis; hyperkalemia as a side effect is extremely rare.^1^ Only two cases reported so far have described hyperkalemia with Daptomycin when used at higher‐than‐recommended doses.^2^, ^3^ Here, we describe a case of hyperkalemia, likely from Daptomycin usage, despite using a low‐dose strength.

## 2 CASE PRESENTATION

A 69‐year‐old Caucasian heavy‐set woman presented to the emergency room with complaints of extreme fatigue, generalized weakness, nausea, dysuria, left flank discomfort, reduced output from the nephrostomy tubes for 3–4 days, fever with chills for 24 h, and a wound with discharge on the right leg stump for 2 days prior to presentation. Her past medical history included diabetes mellitus type 2, hypertension, peripheral vascular disease status post bilateral below‐knee amputations, history of right ovarian cancer with diffuse metastasis status post total abdominal hysterectomy with bilateral salpingo‐oophorectomy and chemotherapy (in remission), bilateral hydronephrosis secondary to extrinsic ureteral compression from ovarian metastatic lesions status post bilateral nephrostomy tubes, chronic kidney disease (CKD) stage 3 with a baseline creatinine around 1.4 mg/deciliter (mg/dL), and an estimated glomerular filtration rate (eGFR) around 40 mL/min/1.73 m^2^ body surface area.

Upon admission, her vitals revealed an elevated blood pressure of 170/70 mmHg, normal oxygen saturation on room air, heart rate of 112 beats per minute, and she was afebrile. Complete blood count (CBC) results showed a hemoglobin level of 9.9 gram (gm)/deciliter (dL), thrombocytosis with a platelet count of 385 × 10 × 9/Liter(L), and a white blood cell count of 12.1 × 10 × 9/L. Basic metabolic panel (BMP) results revealed sodium levels of 140 millimole/ Liter (mmoL/L) (after correction for hyperglycemia), potassium levels of 4.6 mmoL/L, serum bicarbonate levels of 18 mmoL/L, an anion gap of 21, blood urea nitrogen (BUN) levels of 21 mg/dL, creatinine levels of 1.4 mg/dL, and eGFR of 40 mL/min/1.73 m^2^ along with elevated serum blood glucose level of 757 mg/dL. Computed tomography (CT) of abdomen‐pelvis with intravenous (IV) contrast revealed the presence of bilateral nephrostomy tubes without hydronephrosis or pyelonephritis and a thick bladder wall suspicious of cystitis.

She was admitted to the intensive care unit (ICU) for diabetic ketoacidosis with suspected sepsis and started on an insulin drip and antibiotics, IV vancomycin and Zosyn. Urgent bilateral nephrostomy tube exchange was performed on Day 2 as the tubes were not draining correctly. Cultures from the nephrostomy tubes grew *Klebsiella*, *Citrobacter*, *Staph aureus*, *Enterococcus*, *Candida albicans*, and *Acinetobacter*. Peripheral blood cultures were negative. Cultures from the stump discharge grew Candida albicans, Staph epidermidis, and *Enterococcus faecalis*. An MRI of the right leg revealed soft tissue edema, fluid collection, and possible signs of early osteomyelitis. Surgical debridement showed necrotic tissue and abscess, after which stump revision was performed. The antibiotic regimen was changed to vancomycin and cefepime based on the cultures from the right leg wound discharge and nephrostomy tube urine. At this point, infectious disease was consulted to assist with antibiotic management, who recommended discontinuing vancomycin and cefepime, switching to Daptomycin at 4 mg/kg every 48 h for 5 days, and IV fluconazole.

**FIGURE 1:**
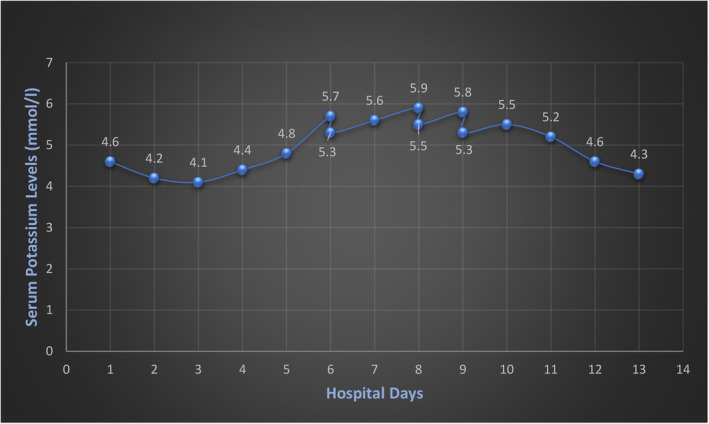
Serum potassium level monitoring during hospitalization.

**FIGURE 2:**
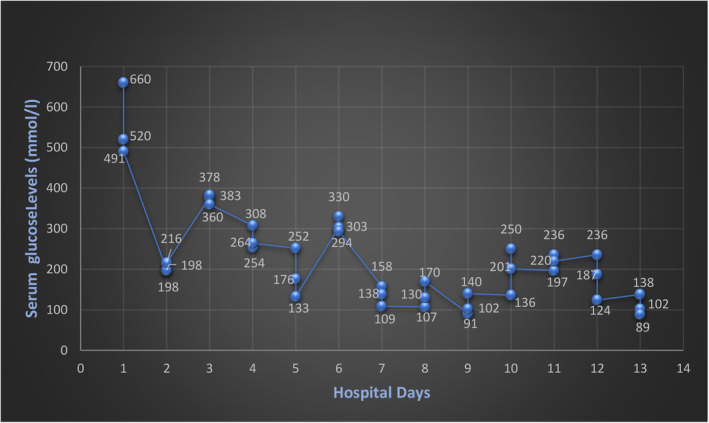
Serum glucose level monitoring during hospitalization.

She developed acute renal failure during the hospital course due to IV contrast exposure, severe volume depletion, and obstructive uropathy. Creatinine has worsened from 1.5 mg/dL on admission to 2.6 mg/dL on day 5. Following nephrostomy tube exchange, correction of diabetic ketoacidosis, and volume replacement, her renal function returned to baseline within 4–5 days. She had sound urine output and stable hemodynamics. Her potassium levels, within the standard limit so far, went from 4.8 mEq/L to 5.7 mEq/L within 24 h without any identifiable cause. Throughout this time, she was not on potassium supplements, ACE inhibitors, or angiotensin receptor blockers, nor she consumed a high‐potassium diet. Her potassium levels continued to rise slowly, reaching 5.9 mEq/L. Hyperkalemia was managed with IV Lasix, Veltassa (patiromer), calcium gluconate, and additional insulin doses. On closer observation, it was noticed that her potassium appeared to worsen after she started receiving Daptomycin. Her potassium levels began to improve 48 h after stopping Daptomycin. Creatinine kinase (CK) levels were checked, as rhabdomyolysis can occur with Daptomycin use. However, our patient did not have rhabdomyolysis (creatinine kinase levels were within normal limits). No further elevation in serum potassium levels was observed during the rest of her hospitalization, suggesting that her hyperkalemia was precipitated by Daptomycin. The correlation of creatinine, potassium level and others are depicted in Table 1. Figures 1 and 2 demonstrate the levels of potassium and glucose levels during the hospital stay, respectively.

**TABLE 1:** Laboratory results from day 1 to 14 during hospitalization.

	Normal Value	Day 1	Day 2	Day 3	Day 4	Day 5	Day 6	Day 7	Day 8	Day 9	Day 10	Day 11	Day 12	Day 13
Serum potassium (mmoL/L)	3.6–5.2 mmoL/L	4.6	4.2	4.1	4.4	4.8	5.7 5.3	5.6	5.9 5.5	5.8 5.3	5.5	5.2	4.6	4.3
Serum glucose (mg/dL)	<140 mg/dL	660 520 491	198 198 216	378 383 360	308 254 264	252 176 133	303 330 294	158 138 109	107 130 170	91 102 140	136 250 201	197 236 220	236 187 124	138 102 89
Serum bicarbonate (mmoL/L)	22–29 mmoL/L	18	18	17	14	15	17	20	22	23	24	21	22	23
Anion gap	7–15	21	12	13	14	13	10	12	9	9	8	9	12	11
Serum creatinine (mg/dL)	0.59–1.04 mg/dL	1.4	1.5	2.0	2.6	2.6	1.9	1.8	1.8	1.5	1.3	1.5	1.5	1.2
eGFR	≥60 mL/min/BSA	40	47	25	19	20	28	30	30	36	41	40	42	45
Daptomycin administered						300 mg		300 mg	Discontinued					
Medications		IV contrast					Patiromer (Veltassa) Lasix Calcium Gluconate Insulin		Patiromer (Veltassa) Lasix Calcium Gluconate Insulin	Patiromer (Veltassa) Lasix				

## 3 DISCUSSION

Daptomycin, a lipopeptide antibacterial agent, is used in treating complicated skin and soft tissue infections and Staphylococcus aureus‐mediated bloodstream infections.^2^ Its activity is limited to gram‐positive organisms, making it an alternative to vancomycin therapy for methicillin‐resistant staphylococcus aureus (MRSA) bacteremia and also joints, articular infections^3^ while being less nephrotoxic than vancomycin.^3^ It is frequently used as an alternative to other anti‐MRSA agents for long‐term outpatient antibiotic therapy due to its convenient dosing and administration.^1^

Daptomycin being bactericidal, acts by forming complexes with cell membrane calcium and phosphatidylglycerol (PG), creating selective pore‐channels in the membrane for cations such as Na+, K+, and other alkali metal ions.^3^ Its mode of action is concentration‐dependent, with the ratio of peak concentration (C max) to minimum inhibitory concentration (MIC) exhibiting the strongest correlation with its activity.^4^ Pharmacokinetic studies indicate that Daptomycin follows a two‐compartment model in plasma with first‐order elimination. It displays linear pharmacokinetics and dose‐proportionality up to 12 mg/kg doses and is predominantly eliminated through the renal route.^4^ The interaction of Daptomycin with bacterial membranes relies on calcium ions, as they neutralize anionic charges and facilitate association with the membrane head groups.^4^ Compared to Vancomycin, gentamicin, and other antimicrobial drugs, Daptomycin is more effective in preventing biofilm formation, adhering to prosthetic devices, and inhibiting colonization of preexisting biofilms.^4^

Commonly reported side effects of Daptomycin include anemia, diarrhea, nausea, vomiting, local injection site reactions, and headache. Although infrequent, Daptomycin can induce myotoxicity, characterized by elevated creatinine kinase levels, myalgias, myositis, weakness, and occasionally rhabdomyolysis.^4^ Hyperkalemia is not a commonly observed side effect of Daptomycin.^2^

Two case reports were found, which were published by Budovich et al., where they described a case of Daptomycin‐induced hyperkalemia in a patient with normal renal function,^2^ and Ibarra et al. which described a case of Daptomycin‐induced hyperkalemia as an early sign of rhabdomyolysis.^3^

Myotoxic effects are more likely at higher doses, such as 8 mg/kg/day, than at lower doses.^3^ Trimethoprim‐Sulfamethoxazole (TMP‐SMX) is a commonly associated antibiotic with hyperkalemia. Combining TMP‐SMX with a high Daptomycin dose could lead to severe hyperkalemia. Therefore, caution should be exercised when using Daptomycin concurrently with other medications known to cause hyperkalemia.^3^ While Bactrim induces hyperkalemia by inhibiting renal potassium elimination, Daptomycin releases intracellular potassium into the serum. The mechanism of rhabdomyolysis involves the formation of membrane pores by Daptomycin polymers intertwining with lipids in muscle cell membranes. Since human cell membranes, including muscle cells, have similar lipid constituents to gram‐positive bacterial membranes, particularly the PG component, high doses of Daptomycin can attach to rhabdo myocyte's lipid membranes, resulting in depolarization, cell lysis, and the subsequent release of intracellular potassium into the blood, leading to elevated serum potassium levels prior to an increase in creatine phosphokinase (CPK) serum levels.^3^ In other words, hyperkalemia should be considered a warning sign for the development of rhabdomyolysis, and dosage adjustments should be made as soon as hyperkalemia becomes apparent.

In our case, she had multiple conditions that could have caused hyperkalemia, such as insulin deficiency (DKA), obstruction of the nephrostomy tubes, tissue breakdown from the stump, thrombocytosis, and acute kidney injury (AKI). Nevertheless, she did not have hyperkalemia due to any of these conditions. All these conditions have been resolved by the time hyperkalemia manifested. The only factor significantly correlated with the onset of hyperkalemia was the timing of Daptomycin administration.

She did not have hyperkalemia when her serum creatinine was as high as 2.6 mg/dL but when her creatinine improved to 1.8 mg/dL and further to 1.5 mg/dL, she had persistenlty elevated serum potassium. This development of sudden hyperkalemia coincided with the introduction of IV Daptomycin rather than her AKI and resolved after discontinuing the drug.

She has CKD 3 at baseline with baseline creatinine around 1.4 mg/dL and Egfr around 40 mL/min to 45 mL/min. She did not have hyperkalemia on admission and at discharge. She did not have hyperkalemia even at post hospitalization follow‐up few months later. This proves that her hyperkalemia is not from her CKD.

Usually hyperkalemia becomes an issue at advanced CKD (that is when Egfr is below 20 mL/min). But our patient's CKD was not at such an advanced stage where she would be at risk for hyperkalemia. Moreover CKD alone does not lead to hyperkalemia unless there is a precipitating event/cause. In our patient's case, Daptomycin was that trigger.

Our patient was on fluconazole which can cause hyperkalemia through adrenal insufficiency. However, she had no signs or symptoms to suspect adrenal insufficiency as her BP was stable, and she had no hyponatremia. There was no evidence of cell lysis based on the patient's average phosphorus level of 4.5 mg/dL and creatine kinase level of 20 units/L. Studies tell us that when used in low doses, Daptomycin can cause potassium to shift from the intracellular space by creating pores in the cell membrane without resulting in complete cell lysis. This mechanism may explain the hyperkalemia observed in our patient without evidence of cell lysis. It is important to note that the patient did not have constipation; instead, she had multiple bowel movements daily, likely because of antibiotics. When the patient developed hyperkalemia, urine sodium, and chloride levels were 69 mEq/L and 61 mEq/L, respectively. These levels were measured while the patient was receiving IVfluids (normal saline). Unfortunately, we did not measure urine potassium levels in our patient.

In our patient, hyperkalemia developed after initiating Daptomycin, and each dose of Daptomycin resulted in a spike in serum potassium levels. Discontinuing Daptomycin led to an improvement in serum potassium levels without any other intervention. This could be attributed to the low dose (4 mg/kg) she was receiving. Daptomycin can induce pore formation in rhabdomyocytes at such low doses, causing intracellular potassium leakage into the serum without triggering frank rhabdomyolysis.

The dosing frequency directly impacts skeletal muscle more than peak plasma concentrations of Daptomycin. Administering the drug less frequently gives time for the repair of damaged myocytes. The current recommendation is to monitor serum CPK concentration at least weekly and more frequently if clinically indicated.^5^ Daptomycin should be given intravenously once daily, as skeletal muscle adverse effects have been observed more frequently with shorter dosing intervals. Daptomycin should be discontinued if patients experience myopathy with CPK elevation or without myopathy but with significant CPK elevations (6.5‐ to 10‐fold upper standard limit). Concurrent use of medications that can potentially cause myopathy, rhabdomyolysis, or CPK elevation should be avoided. For example, consideration should be given to suspend the use of statins or cyclosporine during Daptomycin therapy.^4^

## 4 CONCLUSION

Our case demonstrates that low doses of Daptomycin can induce hyperkalemia without causing rhabdomyolysis. Our patient received a dose every 48 h. We recommend that in patients with CKD or AKI, the dosing of Daptomycin should be even less frequent due to its renal elimination, all potential hyperkalemia‐causing drugs should be avoided, and close monitoring of serum potassium and CPK should be practiced. More reports must be published to inform clinicians of this rare association between Daptomycin and hyperkalemia.

## AUTHOR CONTRIBUTIONS

**Praveen Errabelli:** Conceptualization; writing – original draft. **Maulik Lathiya:** Data curation; writing – review and editing. **Sasmit Roy:** Writing – review and editing.

## FUNDING INFORMATION

The authors declare no funding for the article.

## CONFLICT OF INTEREST STATEMENT

The authors declare no conflicts of interests.

## CONSENT

Written informed consent was obtained from the patient to publish this report in accordance with the journal's patient consent policy.

## Data Availability

Data supporting this study are included within the article.
